# Human cellular microRNA hsa-miR-29a interferes with viral nef protein expression and HIV-1 replication

**DOI:** 10.1186/1742-4690-5-117

**Published:** 2008-12-23

**Authors:** Jasmine K Ahluwalia, Sohrab Zafar Khan, Kartik Soni, Pratima Rawat, Ankit Gupta, Manoj Hariharan, Vinod Scaria, Mukesh Lalwani, Beena Pillai, Debashis Mitra, Samir K Brahmachari

**Affiliations:** 1 Institute of Genomics and Integrative Biology (IGIB) CSIR, Mall Road, Delhi-110007, India; 2 National Centre for Cell Science (NCCS), University of Pune campus, Ganeshkhind, Pune-411007, Maharashtra, India

## Abstract

**Background:**

Cellular miRNAs play an important role in the regulation of gene expression in eukaryotes. Recently, miRNAs have also been shown to be able to target and inhibit viral gene expression. Computational predictions revealed earlier that the HIV-1 genome includes regions that may be potentially targeted by human miRNAs. Here we report the functionality of predicted miR-29a target site in the HIV-1 *nef *gene.

**Results:**

We find that the human miRNAs hsa-miR-29a and 29b are expressed in human peripheral blood mononuclear cells. Expression of a luciferase reporter bearing the nef miR-29a target site was decreased compared to the luciferase construct without the target site. Locked nucleic acid modified anti-miRNAs targeted against hsa-miR-29a and 29b specifically reversed the inhibitory effect mediated by cellular miRNAs on the target site. Ectopic expression of the miRNA results in repression of the target Nef protein and reduction of virus levels.

**Conclusion:**

Our results show that the cellular miRNA hsa-miR29a downregulates the expression of Nef protein and interferes with HIV-1 replication.

## Background

MicroRNAs (miRNAs) are naturally occurring small RNA molecules that modulate gene expression by binding to partially complementary target sites usually located in the 3'UTR of protein coding transcripts[1]. They have been implicated in biological functions like tissue differentiation, establishment of cell fate during development, apoptosis and oncogenesis [2-5]. The cellular miRNA, hsa-miR-32 has been shown to directly interfere with the replication of primate foamy virus in HeLa cells and to reduce viral RNA levels[6]. Another cellular miRNA, miR-122a, involved in cellular stress response and modulated by interferon beta, can also influence the susceptibility to Hepatitis C virus [7-9]. Earlier, we had predicted sites in the HIV-1 genome that can be potentially targeted by human encoded miRNAs using consensus target prediction, and we had proposed the possibility that the cellular levels of these miRNAs may determine disease progression following HIV-1 infection[10]. Here we experimentally confirmed our computational predictions by demonstrating that the expression of specific cellular miRNAs can reduce target protein expression and HIV-1 replication in cultured human cells.

**Figure 1:**
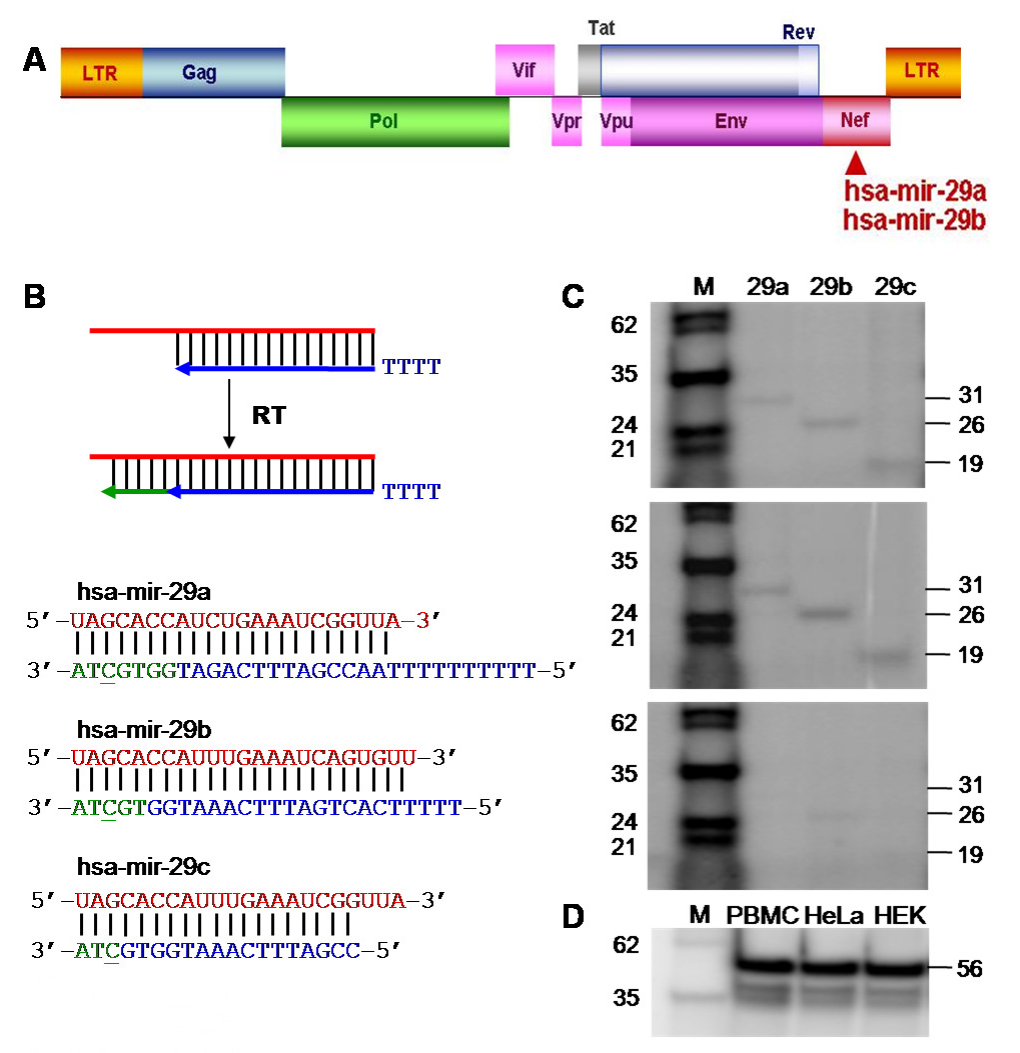
**Detection of hsa-miR-29a, b, c in different cell types**. (A) Schematic representation of the HIV-1 genome. Potential target sites complementary to hsa-miR-29a and b are marked with an arrow. (B) Schematic representation of the method of detection and the oligonucleotides used to capture the miRNA(s). The oligonucleotides (blue) prime sequence specific extension (green) of each miRNA due to differences at the 3' end of the oligonucleotide-miRNA hybrid. The extension product is radiolabeled by the introduction of alpha-P^32^-dCTP into the product at the positions underlined. The T tail of varying lengths at the 5' end is used to improve the resolution of the products. (C) Expression of hsa-miR-29a, b, c in PBMC (upper panel), HeLa (middle panel) and HEK293T (lower panel) cells. HEK293T cells show much lower expression level of hsa-miR-29a and b than PBMCs or HeLa cells. (D) U6 RNA was detected using a similar approach to establish equal input of RNA. Product sizes (nucleotides, nt) including length of T tails are indicated on the right. M: radiolabeled marker.

We used a consensus approach to predict targets in the HIV-1 genome for all human miRNAs. Five miRNAs (hsa-miR-29a, 29b, 149, 324–5p and 378) that were prioritized from the predictions by miRanda[11] and further refined using RNAhybrid[12], MicroInspector[13], and DIANA-MicroT[14], showed putative targets in four HIV-1 genes. These included two miRNAs of highly related sequence (hsa-miR-29a and hsa-miR-29b) that could potentially bind to the region coding for the accessory protein, Nef of HIV-1 (Fig. 1A). The predicted target site of hsa-miR-29a and 29b, located 407 bases into the *nef *transcript, is highly conserved in the sequences from all clades of HIV-1 (A, B, C, D, F and H), with the exception of the outlier group clade O (see Additional file 1). Previous studies have shown that Nef is critical to the progression of HIV-1 infection [15]. Nef is expressed early during the HIV-1 life cycle; and it represses CD4, promotes the release of infectious HIV virions[16] and establishes a persistent HIV-1 infection. The HIV-1 variant found in a cohort of individuals who failed to show signs of disease manifestation even after 10 years of virus infection harbored a deletion in *nef*. *Nef *deleted viruses thus fail to produce symptoms of acquired immunodeficiency syndrome (AIDS). This is consistent with recent studies that Nef is necessary for efficient viral replication and pathogenesis[17]. As *nef *overlaps with the 3' long terminal repeat (LTR), the inhibition of Nef may also lead to transcriptional repression by interference of the 3'LTR function[18]. Furthermore, miRNA N367 which has been shown to inhibit Nef has also been reported to downregulate the transcription and replication of HIV-1 [19]. The importance of *nef *in HIV-1 infection prompted us to experimentally test the anti-HIV-1 potential of hsa-miR-29a and 29b.

## Results and discussion

To address whether the appropriate miRNAs are expressed in cells susceptible to HIV-1 infection, we first tested for the presence of hsa-miR-29a and 29b in PBMCs isolated from healthy volunteers. We also probed for hsa-miR-29c, as this novel sequence-related miRNA has been added to the hsa-miR-29 family since our original prediction (Fig. 1B). Hwang et al. have earlier reported that miR-29c could not be detected in HeLa cells[20]. However, we detected mature hsa-miR-29a, b and c in PBMCs, using primer extension of species specific probes against 29a, b and c (Fig. 1C, upper panel). To identify cells suitable for overexpression of these miRNAs or their depletion using anti-miRNA molecules, we determined the endogenous levels of hsa-miR-29a, b and c in a variety of cell lines. The epithelial derived HeLa cell line has been used widely for reporter assays. We found that HeLa cells expressed 29 a, b and c miRNAs (Fig. 1C, middle panel) at levels comparable to PBMCs. HEK293T cells used extensively for HIV-1 single cycle replication studies, on the other hand, did not show detectable levels of miRNA (Fig. 1C, lower panel). However, in later experiments we could observe expression of hsa-miR-29a, b in HEK293T cells using miRNA specific qRT-PCR Taqman assays. HEK293T cells, therefore, appeared to be appropriate for artificially expressing the miRNAs and for studying anti-HIV-1 potential in single cycle replication experiments. In addition, cell line specific miRNA expression profiling studies have reported that 29 a, b and c are expressed in Jurkat cells [21].

**Figure 2:**
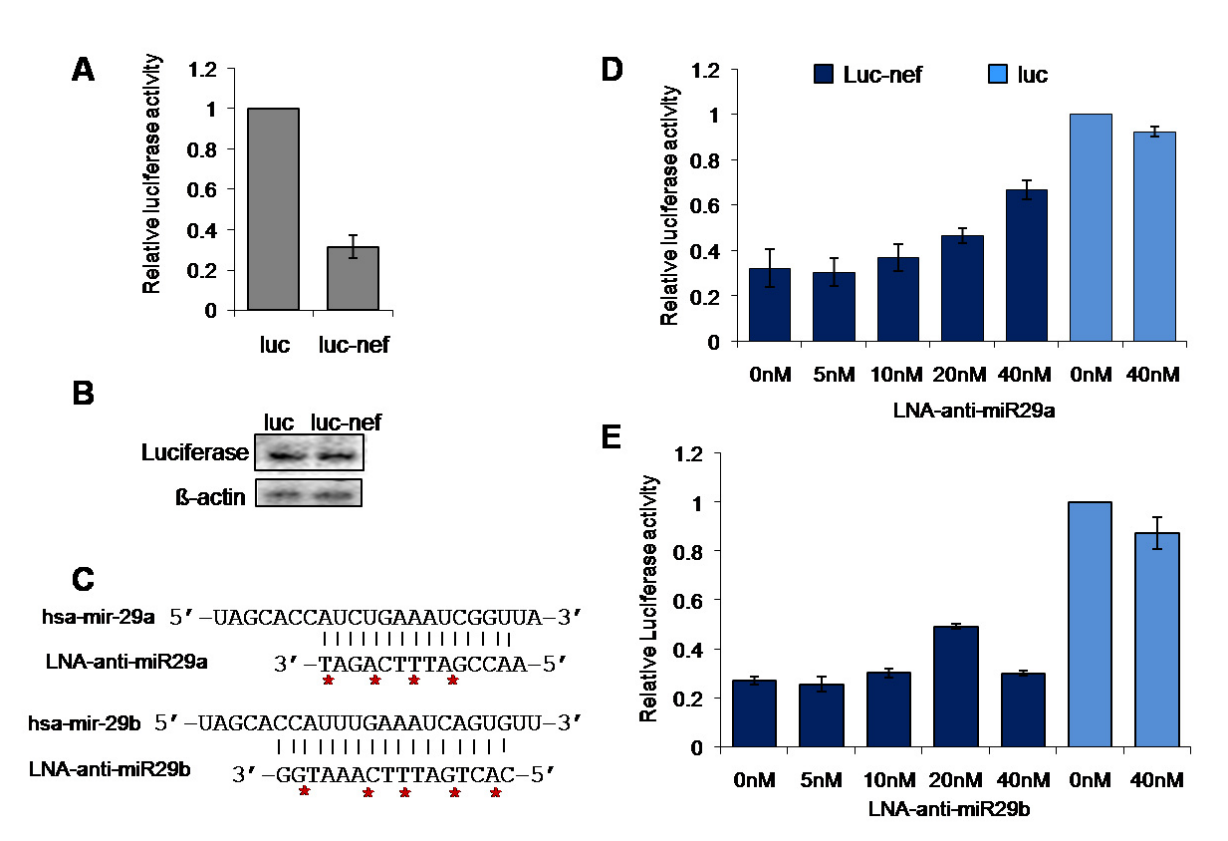
**Nef target region downregulated reporter (luciferase) activity at a post-transcriptional level (A and B) while LNA modified anti-miRNA restored reporter activity (C, D and E)**. (A) A construct containing the *nef *target region cloned into the MCS (3'UTR) of pMIR-REPORT™ Luciferase vector was co-transfected into HeLa cells along with pMIR-REPORT™ β-gal vector. After 24 h, luciferase activity was measured and normalized to beta-galactosidase levels. Relative Luciferase activity was calculated with respect to cells transfected with pMIR-REPORT™ vector alone. Data represent mean ± SEM of three independent experiments performed, each in triplicates. (B) Post-transcriptional regulation of Nef. Northern blot using luciferase probe to show transcript levels of luciferase in luc and luc-nef transfected HeLa cells. β-actin was used as a loading control (lower panel). (C) Design of LNA-modified anti-miRNA molecules for hsa-miR-29a and 29b. Red asterisks indicate positions of LNA-modification in the backbone of the anti-miRNA molecule. (D and E) LNA-modified anti-miRNA against hsa-miR-29a (D) and hsa-miR-29b (E) restored reporter activity from the luc-nef transfected cells. Varying concentrations (0 nM–40 nM) of LNA-modified anti-miRNA molecules were co-transfected with luc-nef clone and control pMIR-REPORT™ β-gal vector into HeLa cells. Luciferase activity was measured after 24 hours and normalized to beta-galactosidase levels. Luciferase activity relative to vector (luc) was plotted. Mean ± SEM from three replicate experiments are shown. LNA, locked nucleic acid; luc, pMIR-REPORT™ vector; and luc-nef, nef target cloned in 3'UTR of pMIR-REPORT™.

Reporter assays using the *nef *target region fused to the 3'UTR of luciferase gene in pMIR-REPORT™ Luciferase (luc) were used next to test the suppressive ability of the cellular miRNAs. The reporter-target fusion construct (luc-nef) was co-transfected with a control beta-galactosidase expression plasmid into HeLa cells which naturally express hsa-miR-29a and 29b. Luciferase activity from the reporter plasmid bearing the target region showed a three fold reduction in expression (Fig. 2A) as compared to control luc-vector transfection. Luciferase RNA levels did not show a significant reduction suggesting that the repression was post-transcriptional (Fig. 2B).

Locked nucleic acid (LNA)-modified anti-miRNA molecules interfere with miRNA function in a highly specific manner[22]. We used LNA-modified anti-miRNA oligonucleotides against hsa-miR-29a and 29b (Fig. 2C) to "knock-down" the cellular miRNAs. Such knock-down should restore reporter activity from the luc-nef fusion construct. Indeed, co-transfection of the luc-nef reporter construct with LNA-modified anti-miR29a and b partially restored the reporter activity in HeLa cells (Fig. 2D and 2E). LNA-modified anti-miR29a was the more effective of the two, restoring reporter activity to 2.5 times that of untreated controls. LNA-modified anti-miR29a and b had no effect on the luciferase vector without the fused *nef *target sequence, supporting the specificity of the LNA-miRNA interactions (Fig. 2D and 2E).

**Figure 3:**
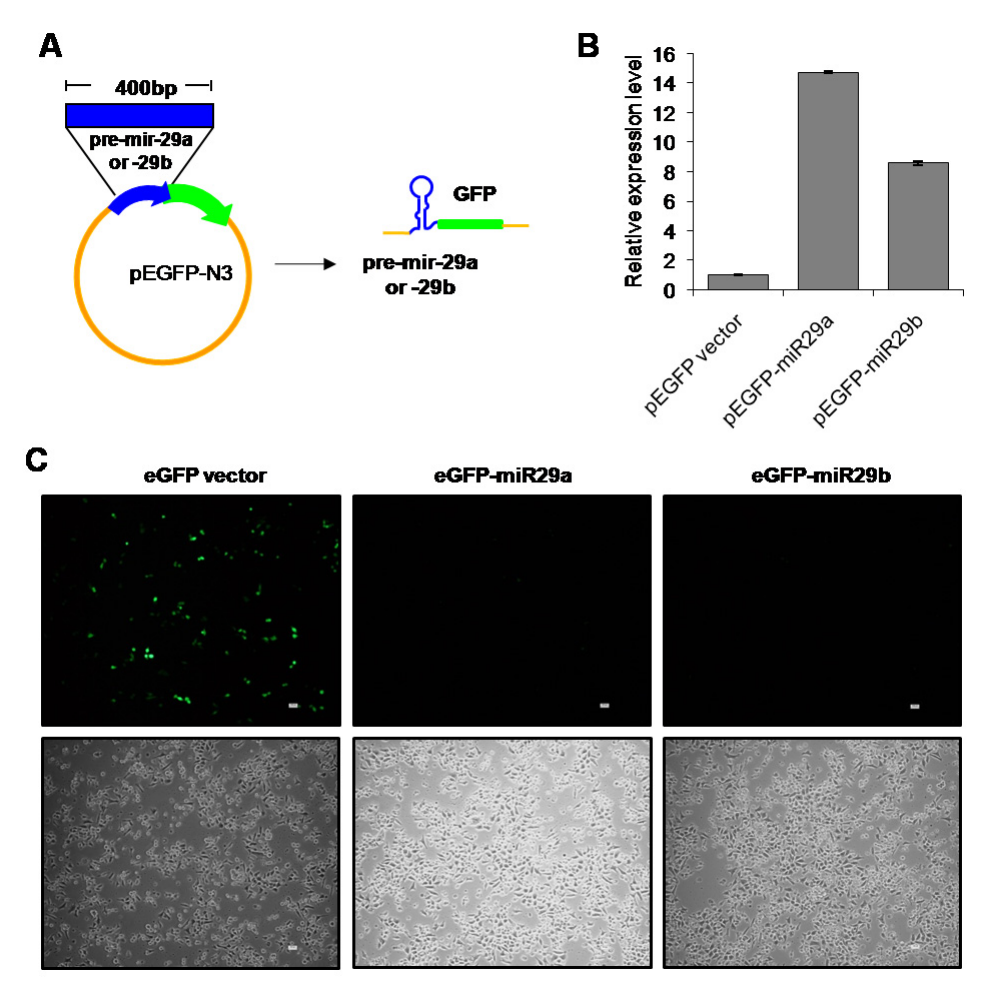
**pEGFP-miRNA construct expressing hsa-mir-29a and 29b**. (A) Diagrammatic representation of pEGFP-N3 vector containing pre-miR29a and 29b. (B) Expression of hsa-mir-29a and 29b confirmed by quantitative real-time PCR. Data show the expression levels of mir-29a and 29b from pEGFP-miR29a and 29b constructs compared to pEGFP-N3 vector. The data represent mean ± S.D. from two independent experiments in triplicates and have been normalized to mir-92 levels. (C) GFP expression from pEGFP-miRNA transfected HEK293T cells. Upper panel represents GFP (+)ve cells, while the lower panel shows total number of cells transfected with pEGFP-N3 (left), pEGFP-miR29a (middle) and pEGFP-miR29b (right), under the 10× objective of a NIKON florescent microscope.

**Figure 4:**
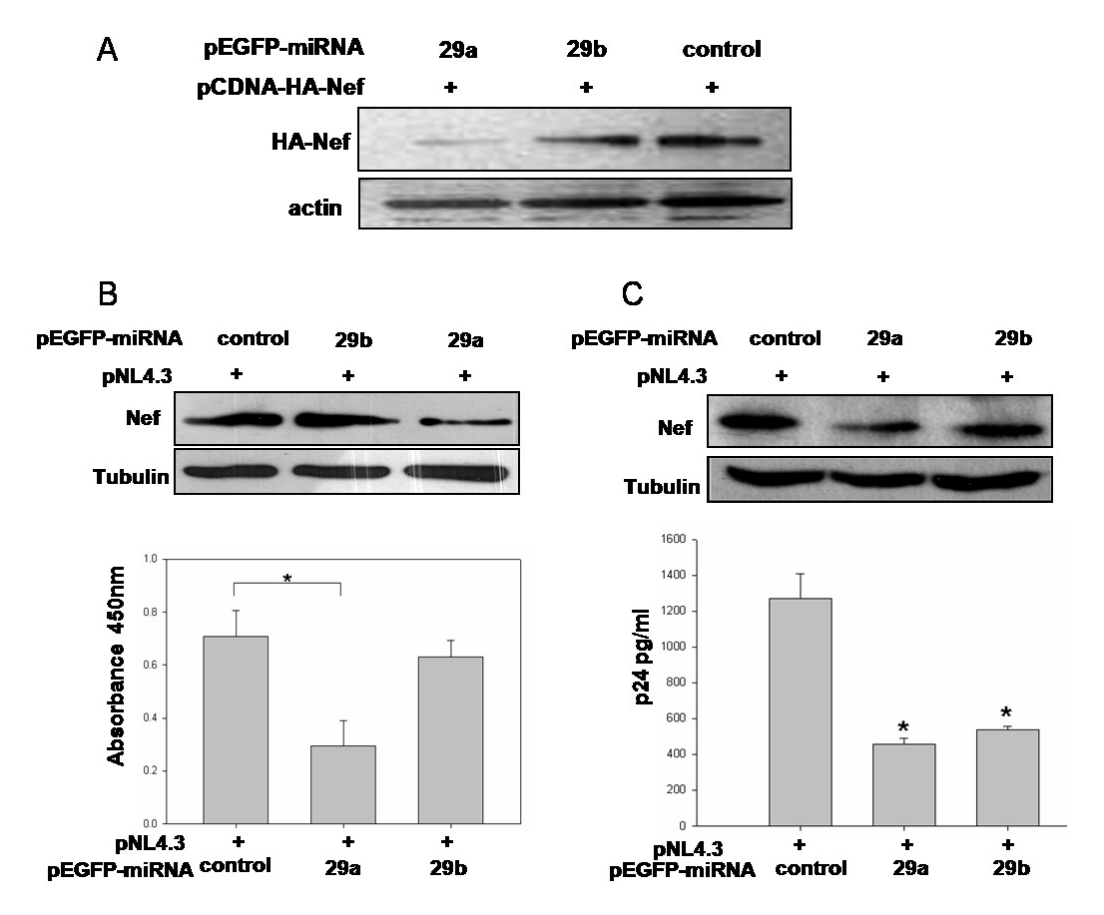
**hsa-mir-29a and b inhibited Nef expression and HIV-1 replication**. (A) Nef expression was inhibited by hsa-miR-29a and 29b. HEK293T cells were co-transfected with miRNA clones or control vector along with pCDNA-HA-Nef using calcium phosphate precipitation. After 36 hours of transfection, cells were lysed and expression of Nef was analyzed by immunoblotting using HA antibody. Immunoreactive actin bands were used as loading control. (B and C) hsa-miR-29a and hsa-miR-29b miRNA clones inhibited virus production in HEK293T (B) and Jurkat cells (C). Cells were co-transfected with miRNA clones or control vector along with HIV-1 molecular clone pNL4.3 (Materials and Methods). Cells were lysed post-transfection and expression of Nef was analyzed by immunoblotting using Nef antibody (upper panels); culture supernatant was used for p24 antigen ELISA (lower panels). Asterisks in (B) represent significant p-value of 0.016 for inhibition mediated by 29a compared to control. The difference observed with 29b is not significant. Asterisks in (C) represent significant p-value of 0.014 and 0.016 for inhibition by 29a and 29b respectively, as compared to control vector.

We next either over-expressed or down regulated hsa-miR-29a and hsa-miR-29b to test the effects of these miRNAs on virus replication. We cloned the pre-miRNA for hsa-miR-29a and hsa-miR-29b into a mammalian expression vector, pEGFP-N3. This construction was designed to express the miRNA under the control of the CMV immediate-early promoter, as a GFP-fusion transcript (Fig. 3A). Expression of the mature hsa-miR-29a and 29b was confirmed by quantitative real-time PCR (Fig. 3B). The inserted pre-miRNA disrupted the 5' UTR region of the EGFP reporter gene. In the absence of splice sites flanking the pre-miRNA, the current model of miRNA processing[23] predicts that miRNA processing would result in a reduced expression of the downstream reporter gene. Indeed, EGFP expression was drastically reduced in the miRNA expressing clones (Fig 3C). We then used these clones to over-express the miRNAs in HEK293T and Jurkat cells transfected with either a Nef expression vector or an HIV-1 molecular clone. In order to study the effect of the miRNA expressed from the EGFP vectors, we co-transfected pre-miRNA-EGFP fusion clones of hsa-miR-29a and b, along with pCDNA-HA-Nef into HEK293T cells and analyzed Nef expression by immunoblotting. As shown in Fig. 4A, both the hsa-miR-29a and 29b expressing clones reduced the expression of Nef, with hsa-miR-29a being highly active. This result indicates that hsa-miR-29a and 29b inhibit Nef expression.

To analyze the effect of these miRNAs on virus replication, we next co-transfected the miRNA-expressing clones with HIV-1 molecular clone pNL4.3 into HEK293T cells. 48 hours post-transfection, the cells were lysed for analysis of Nef expression, and the cell supernatant was used for HIV-1 p24 antigen capture ELISA to quantitatively assess virus production. Over-expression of hsa-miR-29a significantly inhibited both Nef expression and virus production, while modest inhibition was observed with hsa-miR-29b (Fig. 4B, upper and lower panels). This data clearly show that hsa-miR-29a inhibits Nef expression and viral replication in HEK293T cells. As T cells are the primary target of HIV-1, we then used human CD4+ T cell line, Jurkat, for analyzing the role of hsa-miR-29a and 29b in virus replication. Jurkat cells were nucleofected with the miRNA-expressing clones with the pNL4.3 viral clone and analyzed for Nef and p24 expression. In Jurkat T cells, both hsa-miR-29a and 29b clones significantly inhibited Nef expression (Fig. 4C, upper panel) and virus production (Fig. 4C, lower panel). These results, taken together, showed that the two human miRNAs not only inhibit Nef expression but also virus replication.

**Figure 5:**
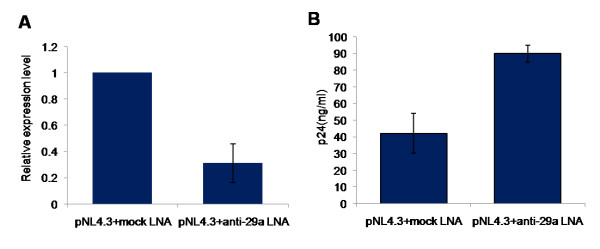
**Cellular hsa-mir-29a inhibits HIV-1 replication**. (A) LNA-modified anti-29a affected the levels of endogenous hsa-mir-29a. LNA-modified anti-29a was co-transfected with pNL4.3 vector. Real time TaqMan microRNA quantification was performed for miR29a. hsa-mir-92 was used as an endogenous control. (B) LNA-modified anti-29a increased HIV-1 replication relative to mock LNA. p24 antigen ELISA was carried out as described previously. Data represent mean ± SEM from two independent experiments performed in triplicates.

In the over-expression studies, hsa-miR-29a consistently showed higher efficacy in reducing viral replication and Nef levels. To investigate the physiological role of cell endogenous hsa-miR-29a on HIV-1 replication, we used LNA modified anti-miR29a to downregulate cellular miR29a in HEK293T cells. As shown in Figure 5A, the LNA modified anti-miR29a resulted in a three fold reduction of the cellular miR29a level. We observed a corresponding increase in virus production, monitored using HIV-1 p24 antigen capture ELISA (Fig. 5B). We used a mock LNA-modified oligonucleotide as a control to control for the specificity of the anti-miR29a oligonucleotide. Anti-miR29a transfected cells showed a two fold increase in viral levels compared to the mock LNA control.

Although Nef was initially reported to be a negative factor for HIV-1, later results from several laboratories including ours [24,25] have found that Nef enhances HIV replication[17]. Interfering with Nef expression is expected to decrease viral replication. Thus, the findings that the inhibition of Nef by ectopic over expression of miR29a and 29b reduced viral replication and that the suppression of endogenous miR29a by anti-miR29a LNA increased viral replication are wholly consistent.

## Conclusion

Accumulating evidence indicates that miRNAs of both viral and host origin may influence host-virus interaction in a variety of ways: as direct modulators of viral replication, as factors affecting viral susceptibility, and as indirect modulators of cellular genes that influence viral propagation [26-29]. In this regard, artificial inhibition of the miRNA processing machinery using siRNAs against Dicer and Drosha has been shown to result in faster replication of HIV-1 in PBMCs[30]. Dicer was also shown to be important in cellular resistance to infection by Vesicular Stomatitis Virus and influenza A virus since cells with Dicer defective alleles or cells with knockdown of Dicer exhibited hypersusceptibility to infection by these viruses [31-33]. A recent report posited that different stages of HIV-1 progression starting with infection followed by the transition from latency to activated replication appears to be associated with discrete expression profiles of cellular miRNAs[34]. That study demonstrated a region common to the 3'UTRs of all HIV-1 transcripts, except *nef*, which is targeted by a cluster of five cellular miRNAs. These miRNAs were suggested to collectively aid in the establishment of viral latency. Based on those observations, one could reason that a panel of miRNA inhibitors might activate latent HIV-1 infection [34]. Compatible with such reasoning, Wang et al. have indeed recently demonstrated that the suppression of anti-HIV-1 miRNAs in monocytes facilitates HIV-1 infectivity, while the increase in macrophages of miRNAs that target HIV-1 inhibited viral replication[35]. Our current results are consistent with the emerging concept that augmenting the expression of cellular anti-viral miRNAs can be a useful strategy in developing anti-HIV-1 therapeutics. In addition expression levels of natural anti-HIV miRNAs may also be useful in studying susceptibility to infection.

miRNAs are nodal molecules in an intricate network of host-virus interactions that form a chain of strategies and counter strategies developed by the virus and the host[26]. Taken together, the range of interactions between the HIV-1 and host cells suggests that miRNAs may be involved in fine tuning the transition from latency to activation, the clearance of latent HIV-1 reservoirs, and the reduction of virion production. Cellular miRNAs with anti-viral roles may have additional roles in host cellular functions. Anti-HIV-1 therapeutics based on the regulation of miRNA levels will have to address how these changes perturb normal cellular homeostasis.

## Methods

### Constructs

pEGFP-N3 (Clontech), pMIR-REPORT™ Luciferase (Ambion) and pMIR-REPORT™ β-gal control plasmid (Ambion) were commercially procured. pEGFP-N3-miR-29a and -29b expressing constructs were prepared as follows: first, PCR amplification of fragments containing pre-miRNA 29a (407 bp) and 29b (417 bp) was carried out using the following primer pairs: 5'-ACAGGATATCGCATTGTTGG-3' and 5'-TATACCACATGCAATTCAG-3' (for 29a) and 5'-CCCAGGCATGCTCTCCCATC-3' and 5'-CATTTGTGATATATGCCACC-3' (for 29b). Next, pEGFP-N3 vector was linearized using restriction enzyme SmaI. Blunt-ends generated were modified by Taq polymerase mediated addition of T overhangs and ligated to the PCR fragments. Luc-nef was constructed as follows: 100 bp sequence containing the *nef *target region was PCR amplified from the HIV-1 genome using primers (restriction site underlined), 5'-CCGACTAGTTTGGCAGAACTACACACC-3' and 5'-CCCAAGCTTGGCCTCTTCTACCTTATC-3', restriction digested with SpeI and HindIII, and cloned into corresponding sites in pMIR-REPORT. All clones were confirmed by sequencing.

### DNA oligonucleotides and LNA modified anti-miRNA

DNA oligonulceotides used for PCR amplification of pre-miRNA and flanking regions, and primer extension based detection of miRNAs were commercially procured (The Centre for Genomic Application, India). Locked nucleic acid-modified oligonucleotides were procured from Proligo.

### Primer extension

Total RNA was isolated from PBMC, HeLa and HEK293T cells using Trizol method (Invitrogen). 5 μg total RNA from each sample was annealed to 10 pmol oligonucleotide designed to capture hsa-miR-29a, -b and -c, respectively followed by extension using radiolabeled P^32^-dCTP and M-MuLV Reverse Transcriptase at 37°C for 30 mins. RNA was denatured and samples resolved on 18% Urea-PAGE. Radioactive bands were detected on Fujifilm FLA2000 phosphorimager. Sequence of the oligonucleotides used is given in Fig 1B, lower panel.

### Northern blotting

10 μg of total RNA from luc and luc-nef tranfected HeLa cells were resolved on 1.5% agarose gel and transferred onto nylon membrane, followed by overnight hybridization with radioactive probe of luciferase prepared as follows: 1.6 kb fragment generated by restriction digestion of pMIR-REPORT™ Luciferase (Ambion) using BamHI and XhoI and radiolabeled using NEBlot™ kit (NEB). β-actin was used as a loading control. Radioactive bands were detected using Typhoon TRIO imager, GE Healthcare.

### Real time PCR

Real time quantification of miRNA expression was performed using TaqMan probes specific for hsa-mir-29a and 29b employing the TaqMan microRNA assays kit (Applied Biosystems) according to manufacturer's protocol. hsa-mir-92 was used as an internal control.

### Cell culture

HeLa, HEK293T and Jurkat cells were propagated in MEM, DMEM and RPMI (Gibco) respectively, supplemented with 10% FBS (Gibco), 1mM sodium pyruvate, 2 mM L-glutamine and 1× Antibiotic (Sigma; broad-spectrum) in 5% CO_2 _and humidified 37°C.

### Transfection and Reporter assay

HeLa cells were co-transfected with pMIR-REPORT (luc or luc-nef), pMIR-REPORT β-gal and varying concentrations of LNA-modified anti-miR-29a or -29b using siPORT *NeoFX *transfection agent (Ambion), according to the manufacturer's protocol. 24 hrs after transfection, cells were lysed in 1× RLB and assayed for luciferase and β-gal activity using luciferase and β-gal assay system (Promega) respectively, according to manufacturer's directions. HEK293T cells were co-transfected with pcDNA-HA-Nef and miRNA clones (pEGFP-N3-miR-29a or -29b) or control vector (pEGFP-N3) using calcium phosphate precipitation. Cells were lysed 36 hrs post-transfection to proceed with immunoblot experiment using Nef antibody.

### Single cycle replication studies

HEK293T were co-transfected with miRNA clones or control vector along with HIV-1 molecular clone pNL4.3 using Lipofectamine 2000. After 48 hrs of transfection, culture supernatant was collected for p24 antigen ELISA (Perkin Elmer Life Science, USA) and cells were lysed for immunoblot experiment using Nef antibody. Similar transfection and assay was also carried out in human CD4+ Jurkat T cells using Amaxa nucleofection system.

## Competing interests

The authors declare that they have no competing interests.

## Authors' contributions

SKB, VS, MH designed the study. JA, SZK, KS, PR, AG performed experiments. BP, DM analyzed the data and SKB, BP, DM wrote the paper.

## Supplementary Material

Additional file 1 Supplementary Figure 1. Tabulated details of target sites of human microRNA 29a on HIV reference sequence and HIV clades. Click here for file
