# Supplementary figures and images for: Human cellular microRNA hsa-miR-29a interferes with viral nef protein expression and HIV-1 replication
Source: Retrovirology. 2008 Dec 23;5:117. doi: 10.1186/1742-4690-5-117 (PMC2635386; doi:10.1186/1742-4690-5-117)

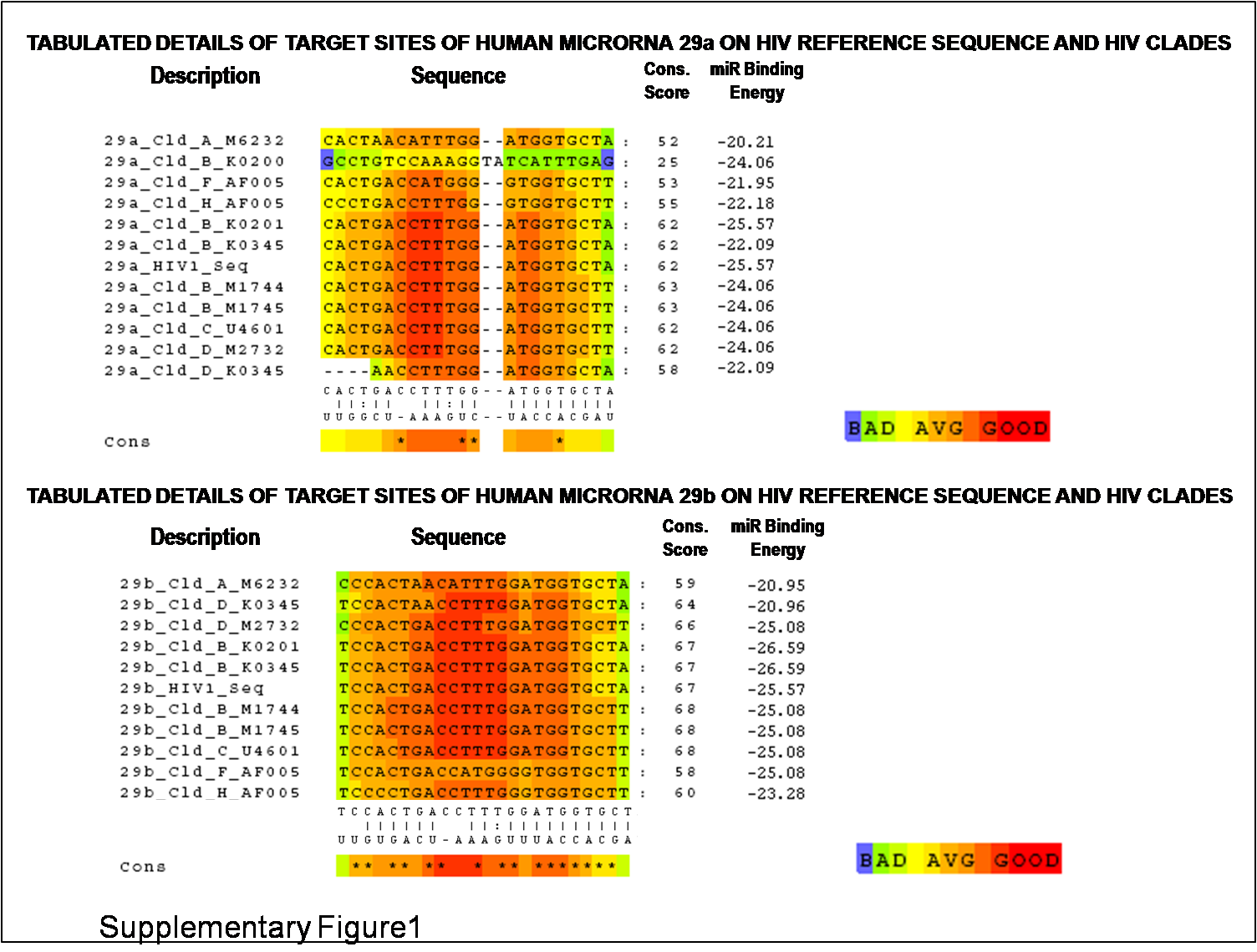

Supplement: Additional file 1 — Supplementary Figure 1. Tabulated details of target sites of human microRNA 29a on HIV reference sequence and HIV clades. [file 1742-4690-5-117-S1.tiff]
